# Polymorphisms of the *ITGAM* Gene Confer Higher Risk of Discoid Cutaneous Than of Systemic Lupus Erythematosus

**DOI:** 10.1371/journal.pone.0014212

**Published:** 2010-12-02

**Authors:** Tiina M. Järvinen, Anna Hellquist, Sari Koskenmies, Elisabet Einarsdottir, Jaana Panelius, Taina Hasan, Heikki Julkunen, Leonid Padyukov, Marika Kvarnström, Marie Wahren-Herlenius, Filippa Nyberg, Mauro D'Amato, Juha Kere, Ulpu Saarialho-Kere

**Affiliations:** 1 Departments of Dermatology, Allergology and Venereology, Institute of Clinical Medicine and Biomedicum Helsinki, University of Helsinki and Helsinki University Central Hospital, Helsinki, Finland; 2 Department of Medical Genetics, University of Helsinki, Helsinki, Finland; 3 Folkhälsan Institute of Genetics, Helsinki, Finland; 4 Department of Biosciences and Nutrition, Karolinska Institutet, Huddinge, Sweden; 5 Research Programme for Molecular Medicine, Biomedicum Helsinki, University of Helsinki, Helsinki, Finland; 6 Department of Dermatology, University of Tampere and Tampere University Hospital, Tampere, Finland; 7 Peijas Hospital, Helsinki University Central Hospital, Vantaa, Finland; 8 Department of Medicine, Rheumatology Unit, Karolinska Institutet, Solna, Sweden; 9 Department of Dermatology, Uppsala University, Akademiska University Hospital, Uppsala, Sweden; 10 Institution for Clinical Sciences, Karolinska Institutet at Danderyd Hospital, Stockholm, Sweden; 11 Department of Clinical Science and Education, and Section of Dermatology, Karolinska Institutet at Stockholm Söder Hospital, Stockholm, Sweden; Dana-Farber Cancer Institute, United States of America

## Abstract

**Background:**

Lupus erythematosus (LE) is a heterogeneous disease ranging from mainly skin-restricted manifestations (discoid LE [DLE] and subacute cutaneous LE) to a progressive multisystem disease (systemic LE [SLE]). Genetic association studies have recently identified several strong susceptibility genes for SLE, including integrin alpha M (*ITGAM*), also known as *CD11b*, whereas the genetic background of DLE is less clear.

**Principal Findings:**

To specifically investigate whether *ITGAM* is a susceptibility gene not only for SLE, but also for cutaneous DLE, we genotyped 177 patients with DLE, 85 patients with sporadic SLE, 190 index cases from SLE families and 395 population control individuals from Finland for nine genetic markers at the *ITGAM* locus. SLE patients were further subdivided by the presence or absence of discoid rash and renal involvement. In addition, 235 Finnish and Swedish patients positive for Ro/SSA-autoantibodies were included in a subphenotype analysis. Analysis of the *ITGAM* coding variant rs1143679 showed highly significant association to DLE in patients without signs of systemic disease (*P*-value  = 4.73×10^−11^, OR  = 3.20, 95% CI  = 2.23–4.57). Significant association was also detected to SLE patients (*P*-value  = 8.29×10^−6^, OR  = 2.14, 95% CI  = 1.52–3.00), and even stronger association was found when stratifying SLE patients by presence of discoid rash (*P*-value  = 3.59×10^−8^, OR  = 3.76, 95% CI  = 2.29–6.18).

**Significance:**

We propose *ITGAM* as a novel susceptibility gene for cutaneous DLE. The risk effect is independent of systemic involvement and has an even stronger genetic influence on the risk of DLE than of SLE.

## Introduction

Lupus erythematosus (LE) is a complex autoimmune disease with variable clinical course and manifestations. Cutaneous lupus erythematosus (CLE) is a heterogeneous disease entity with manifestations primarily confined to the skin. CLE can be subdivided into chronic cutaneous lupus erythematosus, of which discoid lupus erythematosus (DLE) is the most common form, subacute cutaneous lupus erythematosus (SCLE), and acute cutaneous lupus erythematosus (ACLE) [1]. ACLE is characteristic for systemic lupus erythematosus (SLE) that manifests with severe organ involvement and the presence of haematological and immunological abnormalities, whereas well demarcated, scarring plaques of the face, scalp and ears are often seen in DLE patients. Mild extracutaneous involvement may be present in 14–27% of patients with DLE [2], while only 5–10% progress to an overt SLE [3]. Lesions typical for DLE are seen in 15–20% of SLE patients [4]. Population-based studies on the prevalence of CLE are rare, but this condition is estimated to be 2–3 times more frequent than SLE [5]. Although the pathogenic mechanisms underlying LE are not yet entirely established, susceptibility is influenced by multiple genetic and environmental factors, including ultraviolet (UV) radiation, certain drugs and oestrogen [5].

A complex genetic component similar to that of SLE may also underlie CLE [3]. The genetic architecture of CLE is poorly understood, but polymorphisms in *HLA* genes, the *TNF-α* promoter and complement molecules have been suggested as strong candidates for CLE susceptibility [6]. We have recently shown that the known SLE risk genes *TYK2* and *IRF5* also associate with CLE [7].

*ITGAM,* a member of the immune complex processing pathway, has been consistently replicated as an SLE susceptibility gene [8]–[11]. The coding variant rs1143679 (R77H) was recently shown to influence the risk of discoid rash in SLE patients [12]. *ITGAM* (*CD11b*, *Mac-1*) encodes the α-chain of α_M_β_2_-integrin (CD11b/CD18 or CR3), a cell surface receptor for multiple ligands, such as complement 3 cleavage fragment and intercellular adhesion molecule-1 (ICAM-1) [13]. The receptor is expressed on neutrophils, macrophages and dendritic cells and is involved in leukocyte adhesion, phagocytosis and regulation of apoptosis [13]. The exact disease aggravating mechanism is not known, but altered interaction with ligands [10] as well as defects in leukocyte trafficking and uptake of apoptotic cells or immune complexes have been suggested [14]. As abnormal removal of apoptotic cells plays an important role in evolving discoid lesions [6], *ITGAM* is a plausible candidate gene not only for SLE, but also for cutaneous DLE.

In the present study, we investigated the role of *ITGAM* in a well-characterised cohort of DLE patients without signs of systemic disease. The disease risk was further compared between DLE and a cohort of SLE patients that was stratified for various clinical subtypes.

## Materials and Methods

### Ethics statement

This study was conducted in accordance with the Declaration of Helsinki Principles. All subjects gave their written informed consent and the study was approved by the Ethical Review Boards of Helsinki and Tampere University Central Hospitals, Finland, and the Regional Human Ethics Committee at the Karolinska University Hospital, the Institutional Ethics Board and the Regional Ethics Board, Sweden.

### Finnish patients and control individuals

Patients from two Finnish cohorts were included in the present study. The recruitment of the case-control cohort and its clinical characteristics have been described recently [15] (Table 1). Altogether 177 DLE (76% women) and 85 SLE (93% women) patients with LE-specific skin manifestations [16] diagnosed by a dermatologist were recruited. The SLE family cohort included 190 probands (94% women) together with their family members (total n = 236) [17]. The diagnosis of DLE was based on generally accepted clinical, histological and immunofluorescence findings [1] and exclusion of SCLE or SLE. In DLE patients without the characteristic discoid rash (n = 14/177 patients) the diagnosis was based on other phenotype-specific manifestations, such as absence of antinuclear and/or double-stranded DNA antibodies, no signs of extracutaneous disease and dermal perifollicular or perivascular mononuclear cell infiltrate [1] as well as careful clinical examination of patients and patient chart review. Furthermore, the histology and location of lesions usually in the head region, the absence of systemic manifestations and negative laboratory findings in these 14 cases supported the diagnosis of DLE rather than SCLE or SLE. All SLE patients fulfilled the revised 1982 ACR classification criteria by Tan et al. [18] for SLE as confirmed by a rheumatologist. A total of 356 anonymous Finnish controls (49% women) comprising of unaffected spouses or common-law spouses of patients and a collection of unrelated healthy individuals were included in the study.

**Table 1:** Clinical and demographic characteristics of the sets of Finnish patient samples in this study.

	Finnish case-control sample		
	DLE (n = 177)	SLE (n = 85)	SLE families (n = 236 patients)
Female	76	93	94
Mean age at onset (range; yrs)	42 (15–77)	36 (8–85)	29 (1–66)
Mean age at diagnosis (range; yrs)	45 (17–77)	40 (13–86)	33 (6–72)
Butterfly rash	11	72	51
Discoid rash	92	44	10
Annular SCLE lesions	1	12	na
Psoriasiform SCLE lesions	1	22	na
Photosensitivity	65	80	69
Mouth ulcers	4	18	18
Arthritis	2	64	83
Renal involvement	1	20	30
Leukopenia	7	37	68
Thrombocytopenia	5	17	16
Elevated antinuclear antibodies	28	98	na
Ro/SSA-antibody positivity^1^	25	66	na
La/SSB-antibody positivity^1^	4	31	na
Double-stranded DNA antibody positivity	9	37	na

1 Because of differences in laboratory methods and reference values, only patients from Helsinki were included.

Na  =  not available.

Percentage (%) of patients with each phenotype is shown except for mean age, which is shown in years (yrs).

### Swedish patients and control individuals

Patients from two separate Swedish cohorts were also utilised in the present study. Swedish patients (n = 91) participating in a study assessing the incidence and prevalence of SCLE in Stockholm in 1996–2002 [19] (Table 2) were included. In brief, patients positive for Ro/SSA-autoantibodies (Ro-positive patients without signs of systemic inflammation  = 21, DLE  = 2, SCLE  = 8, SLE  = 31, Sjögren's syndrome [SS]  = 23, undifferentiated connective tissue disease [UCTD]  = 6) reported their occurrence of photosensitivity and skin symptoms in a questionnaire. Patients were examined clinically (KP and FN) at the Department of Dermatology, Danderyd Hospital, Stockholm, Sweden. Clinical data were completed based on patient history and medical records. The diagnoses of DLE and SCLE were based on the clinical and histopathological features [1]. The diagnoses of SLE and SS were based on the revised 1982 ACR [18] and the revised European criteria [20], respectively. A diagnosis of UCTD was based on signs and symptoms suggestive of a connective tissue disease and the presence of antinuclear antibodies at two different occasions [21]. Ro-autoantibodies were tested in accredited laboratories in Stockholm using enzyme-linked immunosorbent assay (ELISA) [19].

**Table 2:** Clinical and demographic characteristics of the Swedish sample sets in this study.

	Swedish case-control sample	
	Connective tissue disease patients (n = 91)^1^	Sjögren's syndrome patients (n = 73)^2^
Female	91	95
Mean age at onset (range; yrs)	58 (18–83)^3^	na
Mean age at diagnosis (range; yrs)	na	52 (20–87)
Butterfly rash	13	na
Discoid rash	19	na
Annular SCLE lesions	14	na
Psoriasiform SCLE lesions	0	na
Photosensitivity	100	na
Mouth ulcers	na	na
Arthritis	5	23
Renal involvement	5	4
Leukopenia	na	18
Thrombocytopenia	na	3
Elevated antinuclear antibodies	74	55
Ro/SSA antibody positivity	100	59
La/SSB antibody positivity	38	38
Double-stranded DNA antibody positivity	13	na

1 Ro/SSA positive patients without signs of systemic inflammation  = 21, DLE  = 2, SCLE  = 8, SLE  = 31, SS  = 23, UCTD  = 6 from the study of Popovic et al. [19].

2 SS patients, data provided by Prof. Marie Wahren-Herlenius, MD, PhD, Dept. of Medicine, Rheumatology Unit, Karolinska Institutet, Solna, Sweden.

3 Mean age at initial testing for Ro/SSA-autoantibodies [19].

Na  =  data not available.

Percentage (%) of patients is shown except for mean age, which is shown in years (yrs).

Another Swedish cohort consisted of patients with SS (n = 73) fulfilling the revised European criteria [20] and attending the Karolinska University Hospital, Stockholm, Sweden during 1998–2008 (Table 2). The diagnosis was verified by a combination of clinical examination by rheumatologists or medical doctors and a questionnaire. Ro/SSA-autoantibodies were detected by an ELISA using recombinant, purified Ro52 and Ro60 antigens [22].

A total of 164 Swedish patients with a diagnosis of a connective tissue disease from two distinct cohorts were thus included in the study. Out of these 164 genotyped patients, altogether 134 individuals were positive for Ro/SSA-autoantibodies and were further utilised to evaluate the role of *ITGAM* in the presence of Ro response. Swedish control samples (n = 295; 90.5% women) were population-based anonymous control individuals from the Epidemiological Investigation of Rheumatoid Arthritis (EIRA) study [23].

### Genotyping

After review of the literature regarding *ITGAM* published prior to the initiation of this study [8]–[10], eleven single nucleotide polymorphisms (SNP) at the *ITGAM* locus showing replicated association to SLE in different populations were selected for genotyping: rs1143679, rs9936831, rs9937837, rs9888879, rs12928810, rs9888739, rs11860650, rs6565227, rs1143678, rs4548893 and rs11574637.

Genotyping was performed according to manufacturer's instructions using Sequenom iPLEX Gold chemistry [24] (Sequenom Inc., San Diego, California, USA). Assay design and genotyping were performed in the genotyping core facility at Karolinska Institutet, Huddinge, Sweden. The average genotyping success rate of each marker was 94% for the Finnish case-control cohort, 96% for the Finnish SLE families and 94% for the Swedish case-control cohort; all markers were in Hardy-Weinberg equilibrium (*P*>0.05) in controls. The markers rs12928810 (only in SLE families), rs6565227 and rs1143678 had success rates below our threshold (<85%) and were thus excluded.

### Statistical analysis

**Figure 1:**
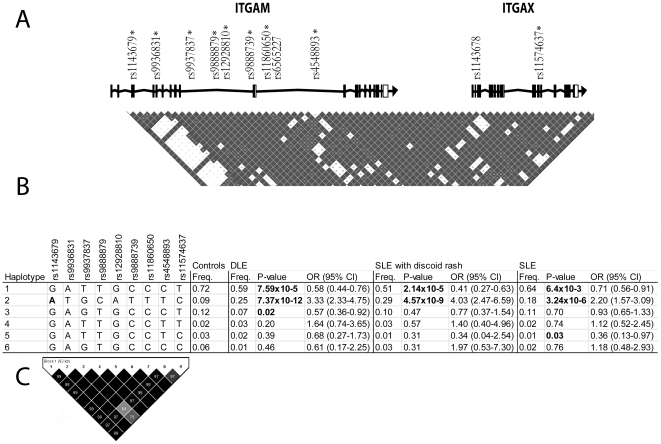
Linkage disequilibrium (LD) patterns across the *ITGAM-ITGAX* locus and haplotype association analysis results. A) LD plot of the genotyped *ITGAM* region on chromosome 16p11 (HapMap CEU data from build 36). Darker colour denotes higher LD (D'). Asterisks indicate single nucleotide polymorphism (SNP) markers genotyped in all datasets in the study. B) Haplotype associations for markers conferring risk for cutaneous DLE, SLE with discoid rash and unstratified SLE. Haplotype 2 is associated with increased, and haplotypes 1 and 3 with decreased risk of cutaneous DLE, SLE with discoid rash, and SLE. Haplotype 2 carries the minor allele A (in bold) of rs1143679, shown in previous studies to tag risk haplotypes for SLE. Significant *P*-values are indicated in bold. The order of SNPs in haplotypes is as follows: rs1143679 - rs9936831 - rs9937837 - rs9888879 - rs12928810 - rs9888739 - rs11860650 - rs4548893 - rs11574637. C) LD plot of the genotyped SNPs in the DLE dataset. Similar LD patterns were observed in unstratified SLE and in SLE patients stratified for discoid rash and renal involvement as well as in Ro/SSA-positive patients (data not shown).

Haploview v. 4.0 [25] was used to study linkage disequilibrium (LD) patterns, estimate haplotypes (Figure 1A–C) and perform association analyses. To increase the statistical power of the association analysis, the sporadic SLE cases (n = 85) were analysed in combination with unrelated probands (n = 190) from the SLE family cohort, thus giving a total of 275 independent Finnish SLE patients and 356 controls for analysis. Allele and haplotype counts were compared between patients and controls by chi-square test, and permutation testing (10 000 iterations) as implemented into Haploview was performed in order to obtain a measure of significance corrected for multiple testing bias. Due the strong LD (Figure 1A) throughout the *ITGAM* locus and thus dependence between markers, traditional Bonferroni correction would likely to be overly conservative. Two-tailed, uncorrected *P*-values are reported in the Results section, and odds ratios (OR) with their respective 95% confidence intervals (CI) were calculated using GraphPad Prism v.4.03 (GraphPad Software Inc., La Jolla, CA, USA).

### Patient stratification for subphenotype analysis

Finnish SLE patients were further stratified by presence of discoid rash (n = 55) and renal involvement (n = 76) defined as fulfilment of the ACR renal criteria [18]. Finnish (n = 101) and Swedish (n = 134) patients positive for Ro/SSA-autoantibodies were first analysed individually and further in combination (n = 235) as a pooled dataset in order to study the association between *ITGAM* and immunological factors [12], [26]. Ro-autoantibodies were selected for detailed investigation, because they may have pathobiological importance in apoptosis and phagocytosis [27] similarly to *ITGAM*, and prognostic value for predicting systemic inflammatory disease [28]. Furthermore, several genetic regions harbouring genes for adhesion molecules have shown evidence of association to DLE in the presence of Ro-autoantibodies [6] and the disease progression may be highly dynamic in Ro-positive patients [29].

### Power calculations

The power to detect association of *ITGAM* to different forms of LE in our sample sets was estimated using the online Power for Association With Error program (http://linkage.rockefeller.edu/pawe/). We assumed a risk allele frequency of 0.185, an OR of 1.70 [11] and alpha 0.05. The average power to detect association of *ITGAM* polymorphisms was 84% in DLE and 91% in SLE. In subphenotype analyses when using reported reference values [12], the power to detect association of rs1143679 was 77% in SLE with discoid rash and 92% in SLE with renal involvement. For Ro-positive Finnish, Swedish and the pooled dataset a power of 70%, 75% and 95%, respectively, was obtained when previously reported risk estimates were assumed [11].

## Results

Nine *ITGAM* SNPs were successfully genotyped in three datasets corresponding to two different LE subgroups (Finnish patients with DLE or SLE) and control individuals. Subphenotype analyses were further performed to investigate the role of *ITGAM* in specific clinical manifestations. These analyses showed that the magnitude of association was strongest in cutaneous DLE (*P*-value ≥3.7×10^−11^; uncorrected P-values are reported), followed by SLE patients with discoid rash (*P*-value ≥3.9×10^−9^) and all SLE patients together (*P*-value ≥8.2×10^−6^) (Table 3). The associations were more modest in SLE patients with renal involvement (*P*-value ≥5.8×10^−5^) (Table 4), in Finnish (*P*-value ≥6.3×10^−6^) and Swedish (*P*-value ≥0.01) Ro/SSA-positive patients as well as in the pooled dataset positive for Ro-antibodies (*P*-value ≥6.4×10^−6^) (Table 5). As hypothesised, the marker rs1143679 (R77H) showed the strongest association in DLE, SLE and SLE with discoid rash (*P*-value range from 4.7×10^−11^ to 8.3×10^−6^) (Table 3). The same marker was observed to reach a more humble, although statistically significant, effect in subphenotype analyses (*P*-value range from 6.4×10^−6^ to 0.04) (Tables 4 and 5), which may mirror the impact of sample size. In addition to rs1143679, the eight other markers studied were observed to associate significantly in Finnish patients (Tables 3, 4, 5), whereas Swedish patients showed borderline association (Table 5). In general, the risk allele frequencies were somewhat higher in DLE, and SLE patients with discoid rash compared to unstratified SLE patients in our study (Table 3) or previous studies, whereas our control individuals had frequencies similar to other control populations of European origin [8], [10], [11]. A similar increase in ORs was also observed, the risk estimates being highest in SLE patients with discoid rash (range 2.46–3.95) and in DLE patients (range 1.65–3.20) followed by unstratified SLE patients (range 1.52–2.14) (Table 3), SLE patients with renal involvement (range 1.82–2.53) (Table 4) and Ro-positive patients (range 1.37–2.03 in the pooled dataset) (Table 5).

**Table 3:** Single marker association results in Finnish DLE, in SLE with discoid rash and in SLE patients.

Marker	Alleles	Associated allele	Controls (n = 356)	DLE (n = 177)			SLE with discoid rash (n = 55)			SLE (n = 275)		
			Freq.	Freq.	*P*-value (corrected)	OR (95% CI)	Freq.	*P*-value (corrected)	OR (95% CI)	Freq.	*P*-value (corrected)	OR (95% CI)
rs1143679	A/G	A	0.10	0.26	4.73×10^−11^ (<0.0001)	3.20 (2.23–4.57)	0.29	3.59×10^−8^ (<0.0001)	3.76 (2.29–6.18)	0.19	8.29×10^−6^ (<0.0001)	2.14 (1.52–3.00)
rs9936831	T/A	T	0.10	0.26	2.14×10^−10^ (<0.0001)	3.03 (2.13–4.31)	0.31	3.99×10^−9^ (<0.0001)	3.95 (2.44–6.40)	0.19	1.32×10^−5^ (<0.0001)	2.08 (1.49–2.90)
rs9937837	G/T	G	0.24	0.34	6.00×10^−4^ (0.004)	1.65 (1.24–2.20)	0.43	1.97×10^−5^ (5.00×10^−4^)	2.46 (1.61–3.76)	0.32	1.20×10^−3^ (0.01)	1.52 (1.18–1.96)
rs9888879	C/T	C	0.10	0.26	7.57×10^−11^ (<0.0001)	3.06 (2.16–4.34)	0.31	3.38×10^−9^ (<0.0001)	3.93 (2.43–6.34)	0.19	1.10×10^−5^ (<0.0001)	2.06 (1.49–2.86)
rs12928810	A/G	A	0.10	0.23	8.03×10^−8^ (<0.0001)	2.77 (1.89–4.07)	na^1^	na	na	na^1^	na	na
rs9888739	T/C	T	0.10	0.25	2.91×10^−10^ (<0.0001)	3.01 (2.12–4.29)	0.30	1.03×10^−8^ (<0.0001)	3.88 (2.38–6.33)	0.18	7.52×10^−5^ (7.00×10^−4^)	1.96 (1.40–2.74)
rs11860650	T/C	T	0.10	0.27	3.71×10^−11^ (<0.0001)	3.13 (2.21–4.43)	0.31	1.10×10^−8^ (<0.0001)	3.81 (2.35–6.18)	0.19	3.36×10^−5^ (1.00×10^−4^)	1.99 (1.43–2.77)
rs4548893	T/C	T	0.15	0.32	1.10×10^−10^ (<0.0001)	2.74 (2.00–3.74)	0.35	2.61×10^−7^ (<0.0001)	3.14 (2.00–4.94)	0.22	9.00×10^−4^ (0.008)	1.64 (1.22–2.21)
rs11574637	C/T	C	0.15	0.30	2.37×10^−9^ (<0.0001)	2.57 (1.87–3.52)	0.37	1.98×10^−8^ (<0.0001)	3.43 (2.19–5.37)	0.22	5.00×10^−4^ (0.004)	1.69 (1.25–2.27)

The frequency of the associated allele in controls and cases is shown, as well as its uncorrected *P*-value and *P*-value corrected for multiple testing (in parentheses) as well as odds ratio (OR) with 95% confidence interval (CI).

Abbreviations: DLE, discoid lupus erythematosus; SLE, systemic lupus erythematosus.

1 The marker has success below the study threshold (<85%) and was excluded from analysis in SLE.

**Table 4:** Single marker association results in Finnish SLE patients with renal involvement.

			Allele associations			
Marker	Alleles	Associated allele	Controls (n = 356)	Patients (n = 76)		
			Freq.	Freq.	*P*-value (corrected)	OR (95% CI)
rs1143679	A/G	A	0.10	0.21	1.00×10^−4^ (0.003)	2.49 (1.54–4.01)
rs9936831	T/A	T	0.10	0.22	1.00×10^−4^ (0.003)	2.43 (1.51–3.89)
rs9937837	G/T	G	0.24	0.35	0.005 (0.03)	1.74 (1.18–2.57)
rs9888879	C/T	C	0.10	0.23	5.83×10^−5^ (0.002)	2.53 (1.59–4.02)
rs9888739	T/C	T	0.10	0.20	0.001 (0.01)	2.22 (1.35–3.65)
rs11860650	T/C	T	0.10	0.23	9.71×10^−5^ (0.002)	2.49 (1.55–3.97)
rs4548893	T/C	T	0.15	0.25	0.002 (0.02)	1.99 (1.29–3.07)
rs11574637	C/T	C	0.15	0.24	0.008 (0.05)	1.82 (1.16–2.83)

The frequency of the associated allele in controls and cases is shown, as well as its uncorrected *P*-value, and *P*-value corrected for multiple testing (in parentheses) as well as odds ratio (OR) with 95% confidence interval (CI). The marker rs12928810 has success below the study threshold (<85%) and was excluded from analysis. Renal involvement is defined as fulfilment of the ACR renal criteria [18].

**Table 5:** Single marker association results in individual Finnish and Swedish patients positive for Ro/SSA-autoantibody as well as in a combined dataset^1^.

Marker	Alleles	Associated allele	Finnish				Swedish				Combined dataset			
			Controls (n = 356)	Patients (n = 101)			Controls (n = 295)	Patients (n = 134)			Controls (n = 651)	Patients (n = 235)		
			Freq.	Freq.	*P*-value (corrected)	OR (95% CI)	Freq.	Freq.	*P*-value (corrected)	OR (95% CI)	Freq.	Freq.	*P*-value (corrected)	OR (95% CI)
rs1143679	A/G	A	0.10	0.22	6.61×10^−6^ (<0.0001)	2.65 (1.71–4.10)	0.10	0.15	0.04 (0.26)	1.62 (1.03–2.54)	0.10	0.18	6.38×10^−6^ (2.00×10^−4^)	2.03 (1.49–2.78)
rs9936831	T/A	T	0.10	0.23	1.35×10^−5^ (<0.0001)	2.54 (1.65–3.90)	0.11	0.16	0.05 (0.36)	1.54 (0.99–2.39)	0.11	0.19	1.54×10^−5^ (2.00×10^−4^)	1.95 (1.43–2.65)
rs9937837	G/T	G	0.24	0.30	0.06 (0.33)	1.40 (0.99–2.00)	0.25	0.31	0.1 (0.53)	1.32 (0.95–1.83)	0.24	0.31	0.01 (0.08)	1.37 (1.07–1.73)
rs9888879	C/T	C	0.10	0.23	6.31×10^−6^ (<0.0001)	2.55 (1.68–3.87)	0.11	0.15	0.05 (0.31)	1.55 (1.00–2.40)	0.10	0.19	7.80×10^−6^ (2.00×10^−4^)	1.97 (1.46–2.66)
rs12928810	A/G	A	0.10	0.18	0.006 (0.0443)	1.96 (1.21–3.19)	0.10	0.16	0.01 (0.07)	1.80 (1.14–2.82)	0.10	0.17	2.00×10^−4^ (0.002)	1.86 (1.34–2.58)
rs9888739	T/C	T	0.10	0.22	1.47×10^−5^ (<0.0001)	2.51 (1.64–3.85)	0.11	0.15	0.08 (0.46)	1.48 (0.95–2.31)	0.10	0.18	3.00×10^−5^ (4.00×10^−4^)	1.91 (1.40–2.59)
rs11860650	T/C	T	0.10	0.23	9.86×10^−6^ (<0.0001)	2.54 (1.66–3.87)	0.11	0.14	0.17 (0.69)	1.37 (0.87–2.16)	0.11	0.18	8.04×10^−5^ (8.00×10^−4^)	1.84 (1.36–2.51)
rs4548893	T/C	T	0.15	0.27	6.96×10^−5^ (0.0009)	2.15 (1.46–3.14)	0.19	0.21	0.43 (0.99)	1.16 (0.80–1.68)	0.17	0.24	8.00×10^−4^ (0.008)	1.57 (1.20–2.04)
rs11574637	C/T	C	0.15	0.25	4.00×10^−4^ (0.0048)	1.99 (1.35–2.93)	0.16	0.21	0.08 (0.48)	1.39 (0.95–2.04)	0.15	0.23	2.00×10^−4^ (0.003)	1.65 (1.26–2.16)

The frequency of the associated allele in controls and cases is shown, as well as its uncorrected *P*-value, and *P*-value corrected for multiple testing (in parentheses) as well as odds ratio (OR) with 95% confidence interval (CI).

1 The Finnish sample set consists of sporadic patients with DLE (n = 36), SCLE (n = 25) and SLE (n = 40) [15]. Ro/SSA information was not available for SLE family probands. The Swedish sample set consists of 21 Ro-positive patients without signs of systemic inflammation, 6 patients with UCTD, 2 DLE, 8 SCLE, 31 SLE, and 66 SS patients.

### Haplotype associations

The haplotype structure of *ITGAM* was further investigated in each set of samples, but this neither improved statistical significance nor yielded any further information (Figure 1 B). One high risk haplotype carrying the minor allele A of rs1143679 (ATGCATTTC; *P*-value  = 7.4×10^−12^, OR  = 3.33, 95% CI  = 2.33–4.75) and two haplotypes with a protective effect (GATTGCCCT; *P*-value  = 7.6×10^−5^, OR  = 0.58, 95% CI  = 0.44–0.76, and GAGTGCCCT; *P*-value  = 1.7×10^−2^, OR  = 0.57, 95% CI  = 0.36–0.92), reaching statistical significance, were found in DLE (Figure 1B). The same risk haplotype with equally strong effect on risk was observed in SLE (OR  = 2.20, 95% CI  = 1.57–3.09), in SLE patients with discoid rash (OR  = 4.03, 95% CI  = 2.47–6.59) (Figure 1B) and in SLE patients with renal involvement (OR  = 2.59, 95% CI  = 1.60–4.21) (data not shown). Finnish and Swedish patients positive for Ro/SSA-autoantibodies analysed either separately or pooled together showed the same haplotype patterns as DLE and SLE patients (data not shown). In line with other studied patient groups, the risk conferring ATGCATTTC haplotype showed evidence of strong association (*P* = 3.0×10^−6^, OR  = 2.08, 95% CI  = 1.52–2.83) within Ro-positive patients when Finnish and Swedish subjects were analysed in combination.

## Discussion

In the present study we have shown that polymorphisms at the *ITGAM* locus increase the risk of cutaneous DLE three-fold compared to healthy control individuals, and the magnitude of the association is five times higher in DLE than in SLE patients. To our knowledge, the current study is the first to address the role of *ITGAM* in a clinically verified cohort of cutaneous DLE patients without signs of systemic disease. We furthermore replicated in our series of SLE patients the recent observation of Kim-Howard et al. [12] that the coding variant rs1143679 (R77H) may influence the risk of discoid rash in SLE.

The marker rs1143679 showed strong association in DLE, in SLE patients with discoid rash and in all SLE patients together. In patient subgroups stratified for renal involvement or Ro/SSA-autoantibody positivity more modest association was observed. The *ITGAM* exon 3 variant rs1143679 was originally identified and replicated in three different populations [10] parallel with GWA studies [8], [9]. To date, its robust association with SLE has been confirmed in a meta-analysis encompassing populations across the world [11], but it has not previously been studied in the cutaneous forms of LE.

Several variants within *ITGAM* and the adjacent *ITGAX* gene (Table 3) were found to associate strongly to DLE and also to SLE with discoid rash. It has, however, been speculated that these multiple association signals are mainly reflecting the strong LD between rs1143679 and nearby markers [11] (Figure 1A), as any haplotype carrying the risk allele A at rs1143679 within *ITGAM* confers risk for SLE [10]. Our haplotype analysis is in line with this (Figure 1B).

Two previous studies have systematically screened several clinical subphenotypes of SLE, including patients with renal and immunological disorders, for association to *ITGAM* [12], [26]. In line with these reports we observed increased risk in SLE patients with renal involvement. We further investigated the relationship between *ITGAM* and immunological disorders in a group of patients positive for Ro/SSA-autoantibodies and found significant association in accordance with findings from a previous study [26]. Possible pathomechanisms underlying these associations have been proposed [12], [26], but not yet established. Interestingly, Ro-autoantibodies and the protein product of the *ITGAM* gene function on the same biological pathways of apoptosis and phagocytosis. It has recently been shown that high Ro52 expression induces apoptosis, and it has been postulated that apoptotic cell opsonisation with Ro-positive sera might rather prevent that than facilitate phagocytosis [27]. However, as Ro-autoantibodies are not exclusive to LE, it is difficult to draw definitive conclusions about this undoubtedly complex relationship between *ITGAM*, Ro-positivity and LE based on observations by us and others [26].

The only *ITGAM* variant for which a functional role has been proposed to date is the previously mentioned rs1143679. This polymorphism results in an amino acid change (R77H) that may alter the structure and function of the receptor protein and further affect its ligand binding activities [10] thus influencing, for instance, ICAM-1 mediated leukocyte recruitment [10], essential in the formation of the inflammatory lesions seen in DLE [6], [14], [18]. Interestingly, *ICAM-1* has been shown to be involved in the pathogenesis of various types of skin disease [30] especially in the presence of Ro/SSA-response [3] and it is upregulated in skin lesions of patients with DLE [30].

*ITGAM* is also interesting in the context of the photosensitivity generally observed in DLE patients [6]. UV-B irradiation initiates a cascade of proinflammatory events in the skin [5], [31]. Upon UV exposure, macrophages expressing *ITGAM* invade the dermis and epidermis [32], [33], leading to a depletion of antigen presenting cells [32], [34]. There is a delay in dendritic cell differentiation in UV-exposed skin [34] and antigenic tolerance is induced [31]. Furthermore, IL-10, a cytokine known to trigger DLE, and discoid rash in SLE [35], is upregulated upon UV exposure. Genetic alterations of *ITGAM* function(s) along this pathway may therefore lead to defects in the suppression of dendritic cell differentiation in combination with high IL-10 production and induce inadvertent immune reactions in DLE patients. Furthermore, UV induces also the production of TNF-α [36], a proinflammatory cytokine that has been, in combination with pronounced IL-10 production, suggested to increase DLE risk [35]. Moreover, TNF-α primes neutrophils to migrate into inflammatory sites through the upregulation of CD11b/CD18 (α_M_β_2_-integrin) [37].

In the present study, a panel of *ITGAM* polymorphisms was specifically tested for association to cutaneous DLE in a well-described cohort of patients that is one of the largest reported. Our study has contributed with *ITGAM* to the short list of genes shown to be associated with DLE. The genetic background of CLE, and that of DLE in particular, is poorly characterised. Furthermore, studies on this disease have been difficult to evaluate and replicate due to low sample sizes non-reporting of risk effect estimates. There are only a few confirmed risk loci, including *HLA* genes, the *TNF-α* promoter and complement factors [6]. However, *TNF-α* shows only modest association [36], [38] and the association may be merely due to the strong LD across the *HLA* region. These sparse data indicate that the genetic background of DLE and other forms of CLE may be very complex and require further studies. Only a few studies have addressed the gender differences in CLE pathogenesis [39] and it is still unclear, what the effect of sex in this disorder may be. Women are known to respond to infection, vaccination and trauma with increased antibody production and a more T helper cell 2 predominant immune response, whereas a T helper cell 1 response and inflammation are usually more severe in men [40]. Thus, it is reasonable to assume that, especially in the function of above mentioned immune system related genes, there would be alterations in the disease mechanisms between female and male subjects. However, investigating this in future studies is a challenging task given the female preponderance of the disease, and carefully matched control individuals are needed as well.

In conclusion, we have demonstrated here a strong novel association between *ITGAM* variants and cutaneous DLE without signs of systemic disease. These variants function independently of the systemic disease and increase substantially the risk of DLE with an effect even higher than in SLE. Our results further strengthen the hypothesis that different forms of LE are not genetically distinct entities even though their clinical course varies. Based on its function(s), *ITGAM* may predispose to DLE through impaired phagocytosis, leukocyte trafficking or immune suppression in UV-exposed skin. The exact mechanisms by which *ITGAM* contributes to the disease pathogenesis and how its expression may be altered in affected DLE skin, warrant further studies.
